# Use of temporary abutment to assess implant osseointegration before final impression: A case report

**DOI:** 10.1002/ccr3.5159

**Published:** 2021-12-04

**Authors:** Nawaf Labban

**Affiliations:** ^1^ Department of Prosthetic Dental Sciences College of Dentistry King Saud University Riyadh Saudi Arabia

**Keywords:** abutment, implant, osseointegration, reverse torque

## Abstract

In implant dentistry, a temporary abutment, either plastic or metal, also called an implant cylinder, is used to construct a provisional restoration. This provisional restoration can be cemented on or integrated with a temporary abutment for a screw‐retained prosthesis. It can be further used as a diagnostic tool to evaluate esthetics and promote tissue healing around implants. After achieving osseointegration of the implant with the adjacent bone and a proper soft tissue profile, both the temporary abutment and the prosthesis can be replaced with permanent ones. In the present case report, a simple technique using a temporary abutment was utilized for the assessment of implant osseointegration before making the final impression. In this study, we discuss the advantages of this method over other methods. It is impossible to verify the stability of the implant at all stages of implant placement; however, the clinical procedure explained in the case report is easy to apply and provides good results.

## Graphical abstract

The torque on a temporary abutment measured before the final impression helps to assess the success of osseointegration and is the least expensive method to avoid extra laboratory and time costs while preparing the crown and in cases of facing failure upon final torquing.

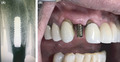

## 1 BACKGROUND

The longevity and success of dental implants are determined by osseointegration, which is in turn dependent on implant stability.^1^ Osseointegration is microscopically defined as a direct contact between the bone and implant detected by light microscopy,^2^ whereas clinically, it is defined as the rigid fixation of an implant to the surrounding bone, maintained during functional load.^3^ After the surgical placement of the implant, wound healing and osseointegration occur in three phases: (1) the inflammatory phase, wherein primary healing occurs through cellular and vascular events, (2) the proliferative phase, where neovascularization occurs, and the woven bone is formed, and (3) the maturation phase, in which ossification of the woven bone occurs. Ossification, which is called remodeling and regeneration of the bone, occurs later.^4^

The stability of the implant is another essential aspect that determines the permanency of the implant. There are two types of stability: primary stability, which is attained by mechanical locking of the implant with the dense cortical bone,^5^ and secondary stability, which is achieved by the remodeling and regeneration of the bone surrounding the implant.^6^ Numerous methods have been proposed to assess the stability of implants at different time points. In this study, the stability of the implant was evaluated postoperatively. Radiography, percussion tests, cutting torque resistance analysis, resonance frequency analysis, modal analysis, and reverse torque tests are some of the methods used to postoperatively assess the stability of implants.^6^ In addition to the methods discussed above, a simple technique that can be used by the clinician to detect the failure of osseointegration is demonstrated in this case report. In implant cases that do not require bone grafting, implant impression is made by the clinician 2–3 months after surgically placing the implant in the bone. Impressions are always made after placing the impression coping, which needs to be screwed onto dental implants using hand torque. Consequently, the implant crown is fabricated in the laboratory and is then intraorally screwed and torqued into place according to the instructions of the manufacturer after clinical adjustments. Although there are no signs of implant failure on the radiograph in some cases, implant osteointegration failure could be discovered during the final torquing of the crown. Unfortunately, at this stage, the laboratory expenses and the clinical time spent on this procedure render this situation very expensive for the dentist. A technique that could be useful in avoiding this scenario is to check for rotation in an implant when a temporary abutment is torqued into place on the implant before making the final impression to guarantee that implant osseointegration has occurred.

## 2 CASE PRESENTATION

**FIGURE 1:**
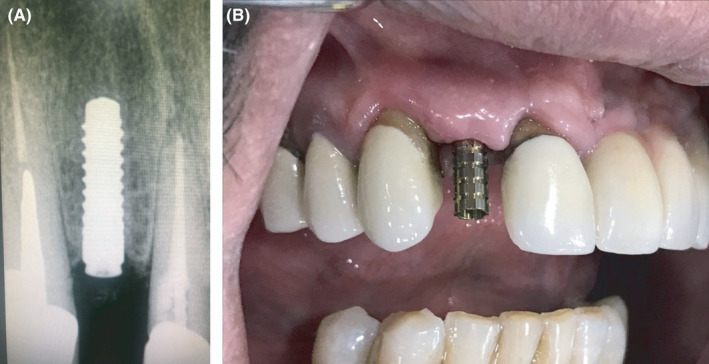
(A) Intraoral periapical radiograph and 1 (B) seating the temporary abutment

**FIGURE 2:**
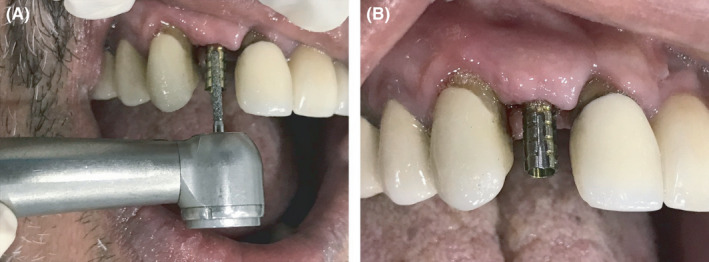
(A) Making groove on the temporary abutment on the mid‐front surface using the handpiece to assess any movement in the abutment during torquing and 2 (B) after creating the groove

**FIGURE 3:**
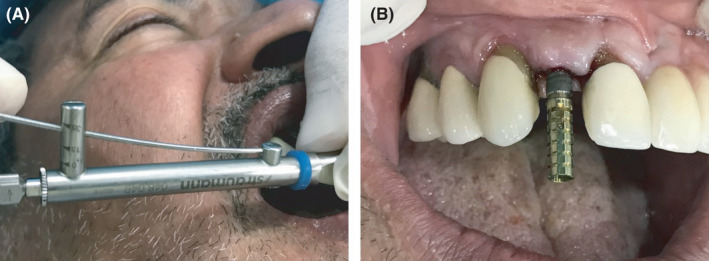
(A) During torquing and 3 (B) failed implant

**FIGURE 4:**
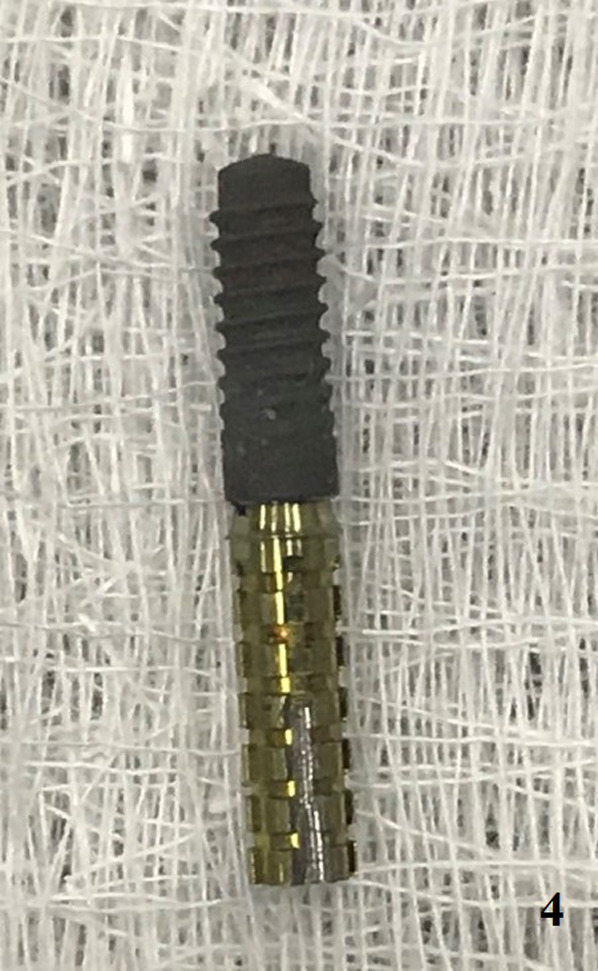
Failed implant

A healthy 55‐year‐old male patient reported to the dental clinic of the author for the replacement of a missing upper right lateral incisor with a dental implant. Preoperative investigations were performed, and undergoing implant treatment was found to be appropriate for the patient. The implant was placed surgically, and no bone graft was required. After 3 months, an intraoral periapical radiograph was taken to detect any signs of osseointegration failure (Figure 1A). The temporary abutment was then seated on the implant using hand torque (Figure 1B). A groove was made on the temporary abutment on the mid‐front surface using a handpiece to observe any movement in the abutment during torquing (Figure 2A and 2B). When a torque was applied (35 Ncm), the implant rotated, indicating failure of the implant to integrate with the osseous tissue (Figure 3A and 3B). Finally, the implant had to be unscrewed from the jaw (Figure 4).

## 3 DISCUSSION

Radiographic assessment is a noninvasive procedure that can be used at all stages of implant placement. However, this method has certain disadvantages. Variations in the bone level around the implant cannot be predicted precisely at 0.1 mm resolution.^7^ The process of obtaining radiographs must be thoroughly standardized to produce a good image. Bone demineralization cannot be observed until the bone is 40% demineralized.^8^ Periapical and panoramic radiographs do not help in evaluating bone loss.^9^ In the present case report, a radiograph was obtained before seating the abutment; however, no signs of implant failure were observed.

The percussion test is a form of a modal analysis used in the health sciences for structural examination. It is based on the concepts of the impact–response theory and vibration and acoustic sciences. Osseointegration is assessed by the sound heard after percussing the implant in the bone. A clear ringing sound is an indication of good osseointegration, whereas a dull sound is an indication of implant failure.^10^ This method of assessment is subjective and based on the experience of the dentist at recognizing the sound, whereas in the present case, the assessment was based on the torque applied while seating the temporary abutment.

The cutting torque resistance analysis was developed by Johansson et al. and improvised by Friberg et al..^11^, ^12^ This method can be used to identify areas of low bone density and measure bone density in osteotomy sites. It also helps in estimating the optimal healing period of the arch after implant placement.^13^ The main disadvantage of the cutting torque resistance analysis is that it can be used only during the surgical phase, and it cannot assess implant stability and the degree of osteointegration postoperatively and before crown placement.^14^ The method discussed in this case report can be used postoperatively but before making the final impression for the final crown placement.

The reverse torque test, proposed by Albrektsson,^15^ assesses osseointegration postoperatively. Although this method is a reliable diagnostic tool to verify osseointegration, it has been observed to cause irreversible plastic deformation in the peri‐implant bone due to the excess load applied during osseointegration. Moreover, this tool cannot assess the degree of osseointegration and can only determine whether or not osseointegration has occurred.^16^

A resonance frequency analysis is a biomechanical method used to measure the bending resonance frequency. This technique is used to evaluate the rigidity of the bone‐implant structure and the depth of implant anchorage in the bone.^17^ However, it cannot be applied for the identification of bone‐implant interface characteristics.^18^, ^19^ All the abovementioned procedures and techniques use expensive and complicated devices. Moreover, all procedures cannot be used to check stability at all the stages of implant placement. However, the clinical procedure explained in the case report is easy to apply and provides good results.

## 4 CONCLUSIONS

The rotation of the implant observed on torquing the temporary abutment before the final impression helps in the assessment of the stability and success of osseointegration. Furthermore, a temporary abutment is the least expensive implant restorative component, which comes with a permanent screw, thus making this procedure economically feasible for dentists. Since the temporary abutment is autoclavable, only the abutment screw needs to be replaced after a specific number of uses, according to the recommendations of the manufacturer for each implant system. Finally, this technique can be used with any other prefabricated abutment available for the implant system utilized for the patient.

## CONFLICT OF INTERESTS

The authors declare that they have no competing interests.

## AUTHOR CONTRIBUTIONS

NF treated the patient, prepared the manuscript, reviewed the literature, and edited and approved the final manuscript.

## ETHICS APPROVAL

Not applicable.

## CONSENT

Written informed consent was obtained from the patient for publication of this case report and any accompanying images. A copy of the written consent is available for review by the editor‐in‐chief of this journal.

## Data Availability

Data sharing is not applicable to this article as no datasets were generated or analyzed during the current study.
